# The Protective Effects of Enalapril Maleate and Folic Acid Tablets against Contrast-Induced Nephropathy in Diabetic Rats

**DOI:** 10.1155/2018/4609750

**Published:** 2018-02-07

**Authors:** Jiantong Hou, Gaoliang Yan, Bo Liu, Boqian Zhu, Yong Qiao, Dong Wang, Ruifeng Li, Erfei Luo, Chengchun Tang

**Affiliations:** Department of Cardiology, Zhongda Hospital of Southeast University Medical School, Nanjing 210009, China

## Abstract

**Background:**

Renal vasoconstriction, oxidative stress, endothelial dysfunction, and apoptosis are the major causes of contrast-induced nephropathy (CIN). The aim of this study was to evaluate the protective effects of enalapril maleate and folic acid tablets on CIN in diabetic rats.

**Methods:**

Thirty-two Sprague-Dawley rats were divided into four groups: CIN (C), CIN + enalapril maleate (CE), CIN + folic acid (CF), and CIN + enalapril maleate and folic acid tablets (CEF). CE, CF, and CEF rats were treated orally with enalapril maleate, folic acid, or enalapril maleate and folic acid tablets, respectively, for 5 days. CIN was induced in all groups followed by analyzed biochemical parameters, oxidative stress markers, endothelial dysfunction parameters, renal histopathology, and TUNEL staining.

**Results:**

Serum creatinine, blood urea nitrogen, and malondialdehyde levels were lower in the CEF group than in the C group. Homocysteine, superoxide dismutase, glutathione peroxidase, and nitric oxide levels were higher in the CEF group than in the C group. Histopathology scores and percentage of apoptotic kidney cells in the CEF group were significantly decreased compared with those in the C group.

**Conclusions:**

These results suggest that enalapril maleate and folic acid tablets have a protective effect against CIN in diabetic rats.

## 1. Introduction

Contrast-induced nephropathy (CIN) is a relatively common and serious complication that can occur after the intravascular administration of iodinated radiographic contrast medium. CIN is defined as an increase in serum creatinine (Scr) of more than 25% or 44.2 mmol/L^−1^ 48 to 72 hours after contrast medium administration without evidence of other causes [1]. Among all causes of hospital-acquired acute kidney injury (AKI), CIN is the third most common and accounts for 11% of all cases [2]. In the general population, the incidence of CIN is estimated to be almost 2% [3]. However, the incidence of CIN in patients with diabetes mellitus (DM) and chronic kidney disease (CKD) can approach 50% [4, 5]. In addition, DM can significantly enhance the occurrence of CIN in patients with renal insufficiency [6]. This life-threatening disease causes prolonged hospitalization and increases the incidence of cardiovascular events and mortality [7]. However, there are still no effective measures to prevent the occurrence of this serious disease. Therefore, the search for more reliable and effective way to treat CIN is critical for patients at high risk of developing CIN.

The pathogenesis of CIN has not been completely explained by a single mechanism, but the development of CIN occurs through a combination of various mechanisms, including acute renal vasoconstriction, contrast medium cytotoxicity to renal tubular cells, oxidative stress, endothelial dysfunction, and apoptosis [8–10]. Homocysteine (Hcy), a sulfhydryl-containing amino acid, is as an intermediate product in the normal biosynthetic pathway of the amino acids such as methionine and cysteine [11]. Hcy levels are significantly elevated in patients with CKD [12]. Hyperhomocysteinemia is directly related to endothelial dysfunction and apoptosis and enhances the occurrence of oxidative stress [13–15]. Enalapril maleate and folic acid tablets are a new compound preparation containing both enalapril and folic acid and are the first approved treatment for hypertension associated with elevated Hcy [16]. Folic acid plays a key role in reducing oxidative stress, improving endothelial function, and preventing apoptosis by reducing Hcy. Moreover, enalapril, a well-known antihypertensive drug, can prevent and treat CIN progression by promoting renal vasodilation [17].

However, no relevant report is currently available regarding whether enalapril maleate and folic acid tablets have a protective effect against CIN. We hypothesized that enalapril maleate and folic acid tablets could prevent CIN due to their renal vasodilation, antioxidative, endothelial-function-promoting, and antiapoptotic effects. In this study, our aim was to investigate the renal protective effects of enalapril maleate and folic acid tablets against CIN in diabetic rats by analyzing biochemical parameters, oxidative stress markers, endothelial dysfunction parameters, histopathological staining, and apoptosis.

## 2. Materials and Methods

### 2.1. Animals

Male Sprague-Dawley rats (300 ± 10 g) were obtained from the Southeast University Animal Center. The animals were housed in a vivarium under controlled photocycle (12 h light/12 h dark) and temperature (22–25°C) conditions with free access to food and water. Our study was approved by the Ethics Review Board for Animal Studies at the Institute of Southeast University, Nanjing, China.

### 2.2. Induction of Diabetes

Twelve hours after fasting, streptozotocin (60 mg/kg; Sigma, St. Louis, MO) was injected intraperitoneally to establish early diabetes mellitus. Blood glucose concentrations were determined after three days, and rats with blood glucose levels over 300 mg/dl were considered to have diabetes mellitus. Glucose levels were tested in rats every day.

### 2.3. Grouping and Drug Intervention

Thirty-two rats were divided into four groups of eight rats: (1) CIN (C), (2) CIN + enalapril maleate (CE), (3) CIN + folic acid (CF), and (4) CIN + enalapril maleate and folic acid (CEF). Rats in the C, CE, CF, and CEF groups received saline, enalapril maleate (Yangzijiang Pharmaceuticals, Jiangsu, China) at 30 mg/kg/day, folic acid (Changzhou Pharmaceutical, Jiangsu, China) at 2.4 mg/kg/day, or enalapril maleate and folic acid tablets (Shenzhen AUSA, Guangdong, China) at 30 mg/2.4 mg/kg/day, respectively, for 5 consecutive days before CIN was induced.

### 2.4. Induction of CIN

CIN was induced by sequential tail vein administration of indomethacin (10 mg/kg), L-NAME (10 mg/kg, twice at 15 and 30 min), and loperamide (10 ml/kg, Bayer, Germany) in rats. Scr concentration was determined according to the CIN definition to evaluate whether the CIN model was successful. The rats were sacrificed 3 days after the induction of CIN. Serum was separated from blood to determine biochemical and endothelial dysfunction parameters. Renal tissues were removed to evaluate oxidative stress markers, histopathology, and apoptosis.

### 2.5. Biochemical Parameters

Blood samples were removed by heart puncture and separated to obtain serum. Scr, blood urea nitrogen (BUN), and Hcy were measured using an auto-biochemistry analyzer AU5800 (Beckman Coulter, USA) within 24 h.

### 2.6. Oxidative Stress Markers

Superoxide dismutase (SOD), malondialdehyde (MDA), and glutathione peroxidase (GSH-PX) levels were determined using experimental kits (Nanjing Jiancheng, China) in renal tissue homogenates.

### 2.7. Endothelial Dysfunction Parameters

Concentrations of serum nitric oxide (NO) and vascular endothelial growth factor (VEGF) were analyzed using commercial kits (Nanjing, Jiancheng, China) according to the manufacturer's instructions.

### 2.8. Renal Histopathology

Kidneys were removed from rats and placed in 4% paraformaldehyde for 24 h, followed by paraffin embedding and sectioning. Renal specimens were then stained with hematoxylin and eosin. We used a renal injury scoring system to assess the degree of kidney damage. Criteria for renal injury were as follows: 0, no injury; 1, minimal tubular epithelial cell edema; 2, hemorrhage and moderate tubular epithelial cell edema; 3, moderate hemorrhage and moderate tubular epithelial cell edema; 4, severe tubular epithelial cell edema and formation of necrosis spots; and 5, intratubular cast presence, severe tubular architecture alteration, and severe tubular necrosis [18]. Evaluations were performed in a blinded manner.

### 2.9. TUNEL Staining

DNA fragmentation in kidney apoptotic cells was determined by TUNEL staining according to the manufacturer's instructions (Roche, Mannheim, Germany). Briefly, kidney samples were paraffin-embedded and cut into 3 *μ*m sections followed by deparaffinization and rehydration. Kidney sections were pretreated with proteinase-K and then incubated with TUNEL reaction mixture for 1 h at 37°C. The number of TUNEL-positive cells was counted in 10 randomly selected nonoverlapping fields in each kidney sample under 400x magnification.

## 3. Statistical Analysis

The data are expressed as the mean ± standard deviation. Data were analyzed using one-way ANOVA followed by Tukey's Multiple Comparison Test using SPSS17.0 (SPSS Inc., Chicago IL, USA). *P* values less than 0.05 were considered statistically significant.

## 4. Results

### 4.1. Biochemical Parameters

**Figure 1:**
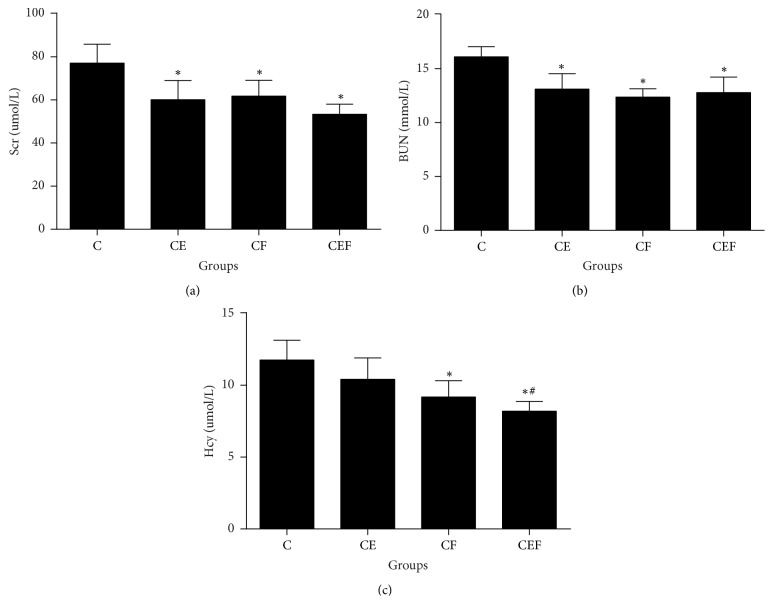
C: contrast-induced nephropathy; CE, contrast-induced nephropathy with enalapril maleate treatment; CF, contrast-induced nephropathy with folic acid treatment; CEF, contrast-induced nephropathy with enalapril maleate and folic acid treatment. Data are presented as the means ± SD (*n* = 8). ^*∗*^*P* < 0.01 versus C; ^#^*P* < 0.05 versus CE.

Scr and BUN levels were significantly lower in the CE, CF, and CEF groups than in the C group (*P* < 0.01). Treatment with folic acid or enalapril maleate combined with folic acid significantly decreased serum Hcy levels (CF and CEF versus C, *P* < 0.01). In addition, serum Hcy levels were lower in the CEF group than in the CE group (*P* < 0.05) (Figure 1).

### 4.2. Oxidative Stress Markers

**Figure 2:**
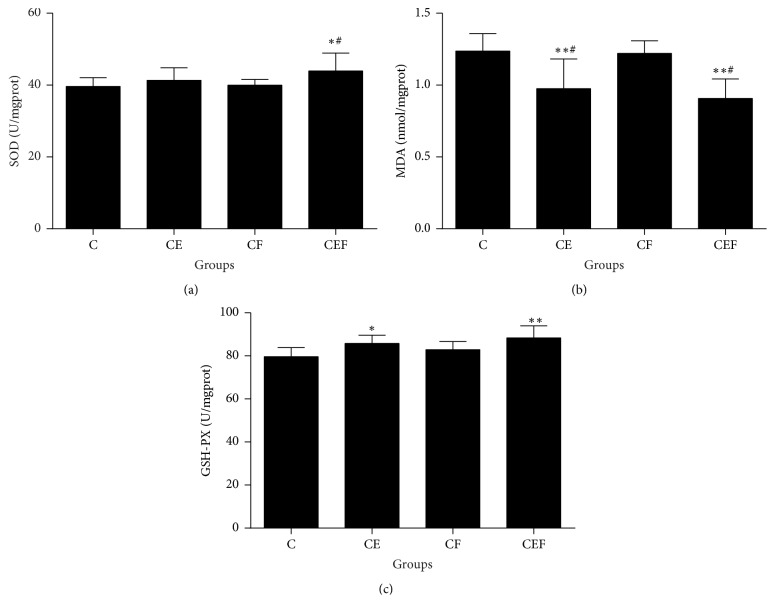
C: contrast-induced nephropathy; CE, contrast-induced nephropathy with enalapril maleate treatment; CF, contrast-induced nephropathy with folic acid treatment; CEF, contrast-induced nephropathy with enalapril maleate and folic acid treatment. Data are presented as the means ± SD (*n* = 8). ^*∗*^*P* < 0.05 versus C, ^*∗∗*^*P* < 0.01 versus C, and ^#^*P* < 0.05 versus CF.

As shown in Figure 2, kidney tissue homogenate SOD levels were higher in the CEF group than in the C and CF groups (*P* < 0.05). MDA levels were lower in the CE and CEF groups than in the C and CF groups (*P* < 0.01 and *P* < 0.05, respectively). GSH-PX levels were higher in the CE and CEF groups than in the C group (*P* < 0.05 and *P* < 0.01, respectively).

### 4.3. Endothelial Dysfunction Parameters

**Figure 3:**
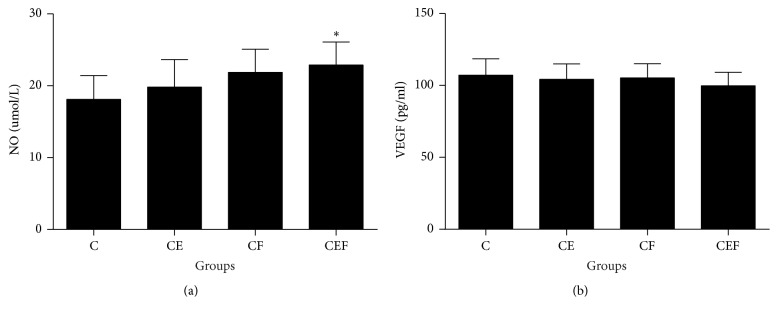
C: contrast-induced nephropathy; CE, contrast-induced nephropathy with enalapril maleate treatment; CF, contrast-induced nephropathy with folic acid treatment; CEF, contrast-induced nephropathy with enalapril maleate and folic acid treatment. Data are presented as the means ± SD (*n* = 8). ^*∗*^*P* < 0.05 versus C.

As shown in Figure 3, serum NO levels were notably increased in the CEF group compared with those in the C group (*P* < 0.05). However, there were no statistically significant differences in serum VEGF between the CE, CF, CEF, and C groups (*P* > 0.05).

### 4.4. Renal Histopathology

**Figure 4:**
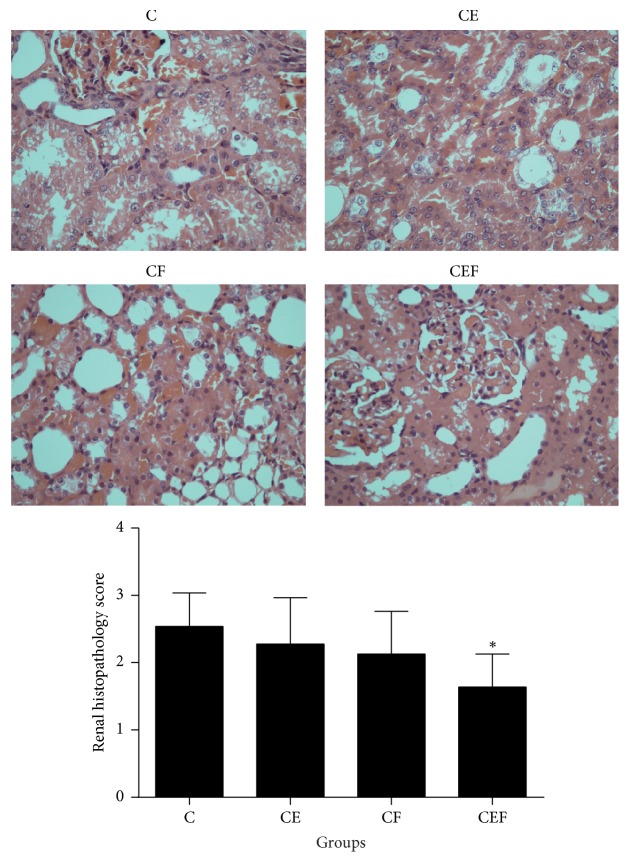
C: contrast-induced nephropathy; CE, contrast-induced nephropathy with enalapril maleate treatment; CF, contrast-induced nephropathy with folic acid treatment; CEF, contrast-induced nephropathy with enalapril maleate and folic acid treatment. Data are presented as the means ± SD (*n* = 8). ^*∗*^*P* < 0.05 versus C.

Figure 4 shows that there were no significant differences in kidney injury scores between the CE, CF, and C groups (*P* > 0.05). However, the renal histopathology score in the CEF group was lower than that in the C group (*P* < 0.05). This suggests that enalapril maleate and folic acid tablets could significantly decrease kidney injury due to contrast medium.

### 4.5. TUNEL Staining

**Figure 5:**
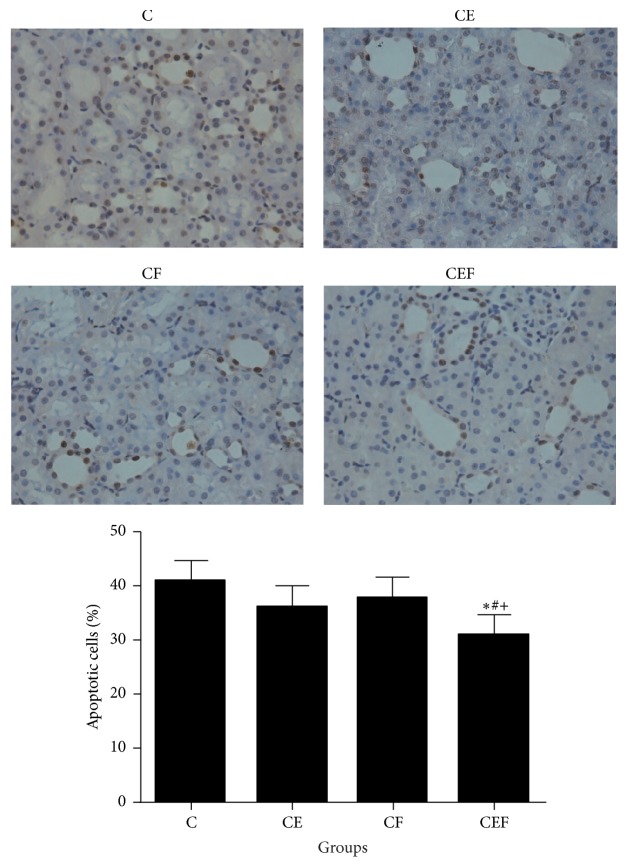
C: contrast-induced nephropathy; CE, contrast-induced nephropathy with enalapril maleate treatment; CF, contrast-induced nephropathy with folic acid treatment; CEF, contrast-induced nephropathy with enalapril maleate and folic acid treatment. Data are presented as the means ± SD (*n* = 8). ^*∗*^*P* < 0.01 versus C, ^#^*P* < 0.05 versus CE, and ^+^*P* < 0.01 versus CF.

As shown in Figure 5, TUNEL staining showed that contrast injection can lead to elevated apoptosis in diabetic rats. The percentage of kidney apoptotic cells was significantly lower in the CEF group than in the C group (*P* < 0.01). In addition, the percentage of kidney apoptotic cells was also decreased in the CEF group compared with those in the CE and CF groups (*P* < 0.05 and *P* < 0.01, respectively). This suggests that enalapril maleate and folic acid tablets could exert an antiapoptotic effect in CIN rats.

## 5. Discussion

CIN has become an increasingly serious iatrogenic complication in clinical practice due to the high morbidity and mortality rates. Although significant efforts to develop prevention and treatment strategies to reduce the incidence and severity of CIN have been made, the results are still unsatisfactory and inconsistent [19, 20]. Therefore, new methods of prevention and treatment for CIN could have significant clinical value. In this study, we demonstrated that enalapril maleate and folic acid tablets can decrease the incidence of CIN in diabetic rats. Our data indicated that pretreatment with enalapril maleate and folic acid tablets could significantly decrease renal injury biomarkers, including Scr and BUN, as well as Hcy. In addition, enalapril maleate and folic acid tablets ameliorated renal function, oxidative stress, endothelial dysfunction, kidney histopathological injury, and apoptosis in CIN rats.

Although the pathogenesis of CIN is not completely understood, CIN is mainly due to acute renal vasoconstriction, oxidative stress, endothelial dysfunction, and apoptosis [8–10]. A previous study also showed that contrast medium can lead to increased release of adenosine, endothelin, and other renal vasoconstrictors, which causes acute renal vasoconstriction and subsequent ischemia-reperfusion injury [21, 22]. Enalapril, an angiotensin-converting enzyme inhibitor (ACEI), can inhibit the synthesis of angiotensin II resulting in vasodilation. It can also enhance the vasodilatory effect of bradykinin to promote vasodilatation further [23]. In this study, we showed that pretreatment with enalapril maleate can significantly decrease Scr and BUN levels. Therefore, we hypothesized that enalapril may improve renal function by promoting vasodilation.

Studies have shown that oxidative stress and the production of reactive oxygen species (ROS) are also important mechanisms in the occurrence and development of CIN [24]. Several studies have shown that angiotensin II promotes the production of ROS by enhancing the synthesis of growth factors and profibrotic cytokines [25, 26]. Enalapril maleate and folic acid tablets may exert an antioxidant effect by inhibiting the synthesis of angiotensin II. Increased serum Hcy is closely related to the occurrence of oxidative stress in local tissues or cells [27]. We recently reported that elevated Hcy prior to percutaneous coronary intervention (PCI) is an important independent risk factor of CIN in acute coronary syndrome (ACS) patients undergoing their first PCI [28]. Hcy can promote the production of ROS by autooxidation and reduction through SOD, GSH-PX, and other antioxidant enzymes [29–33]. In agreement with previous studies, we found that pretreatment with enalapril maleate and folic acid tablets could increase renal SOD and GSH-PX levels and decrease renal MDA levels in CIN rats. We speculated that the renal protective effect of enalapril maleate and folic acid tablets can be attributed to the reduction of oxidative stress.

Endothelial dysfunction is also one of the mechanisms behind the occurrence and development of CIN. A previous study showed that high levels of Hcy can lead to ROS generation and endothelial cell apoptosis by the induction of endoplasmic reticulum stress [34]. Piceatannol can protect endothelial cells against Hcy-induced endoplasmic reticulum stress, oxidative stress, and apoptosis. A previous study showed that autooxidation of Hcy is a major mechanism responsible for endothelial dysfunction that results in lipid peroxidation and generates ROS in endothelial cells [35]. NO and VEGF are important markers of vascular endothelial function [36]. Elevated Hcy levels reduce the bioavailability of NO, which leads to vascular endothelial dysfunction [37]. In this study, NO levels were notably increased in the CEF group compared with those in the C group. This suggests that enalapril maleate and folic acid tablets play a protective role in the kidney via improvement of vascular endothelial cell function.

Apoptosis is also an important factor in the occurrence of CIN, and insufficient folic acid can cause endoplasmic reticulum stress and apoptosis in pancreatic islet *β* cells [38]. This may be related to folic acid deficiency leading to elevated Hcy levels. A previous study showed that Hcy-induced apoptosis of endothelial cells may occur through the accumulation of ROS and the inhibition of NO synthesis [15, 39, 40]. In the present study, we observed that enalapril maleate and folic acid tablets could significantly reduce the percentage of kidney apoptotic cells in CIN rats. Enalapril maleate and folic acid tablets exerted an antiapoptotic effect, which may have occurred through the reduction of Hcy.

Hyperhomocysteinemia can induce the occurrence of oxidative stress, endothelial dysfunction, and apoptosis, which is the important pathogenesis of CIN. Enalapril maleate and folic acid tablets are a new compound preparation containing both enalapril and folic acid that can inhibit angiotensin II synthesis while reducing serum Hcy to exert renal vasodilatory, antioxidant, antiapoptotic, and endothelial function-promoting effects. This compound preparation will further exert renal protection and provide new ideas for the future treatment of CIN in clinical practice.

Several potential concerns and limitations need to be mentioned here. First, the sample size used in this study is too small. CIN model animals can not completely simulate CIN patients in clinical practice. Second, we did not attempt to measure inflammation, apoptosis protein markers, or angiotensin II levels in CIN rats due to financial constraints. However, the potential protective effects of enalapril maleate and folic acid tablets on inflammation in CIN need to be determined. Third, we did not design different doses of enalapril maleate and folic acid tablets for the treatment of CIN. CIN is still an unresolved problem in clinical practice [41, 42], and further animal and clinical studies are needed to determine the efficacy and safety of enalapril maleate and folic acid tablets in CIN prevention and treatment.

## 6. Conclusions

Enalapril maleate and folic acid tablets attenuated renal injury in a CIN rat model by exerting renal vasodilatory, antioxidation, antiapoptotic, and endothelial function-promoting effects. Our findings show that enalapril maleate and folic acid tablets could successfully treat CIN.
